# Herbivory, Connectivity, and Ecosystem Resilience: Response of a Coral Reef to a Large-Scale Perturbation

**DOI:** 10.1371/journal.pone.0023717

**Published:** 2011-08-25

**Authors:** Thomas C. Adam, Russell J. Schmitt, Sally J. Holbrook, Andrew J. Brooks, Peter J. Edmunds, Robert C. Carpenter, Giacomo Bernardi

**Affiliations:** 1 Coastal Research Center, Marine Science Institute, University of California Santa Barbara, Santa Barbara, California, United States of America; 2 Department of Ecology, Evolution and Marine Biology, University of California Santa Barbara, Santa Barbara, California, United States of America; 3 Department of Biology, California State University Northridge, Northridge, California, United States of America; 4 Department of Ecology and Evolutionary Biology, University of California Santa Cruz, Santa Cruz, California, United States of America; University of Western Australia, Zimbabwe

## Abstract

Coral reefs world-wide are threatened by escalating local and global impacts, and some impacted reefs have shifted from coral dominance to a state dominated by macroalgae. Therefore, there is a growing need to understand the processes that affect the capacity of these ecosystems to return to coral dominance following disturbances, including those that prevent the establishment of persistent stands of macroalgae. Unlike many reefs in the Caribbean, over the last several decades, reefs around the Indo-Pacific island of Moorea, French Polynesia have consistently returned to coral dominance following major perturbations without shifting to a macroalgae-dominated state. Here, we present evidence of a rapid increase in populations of herbivorous fishes following the most recent perturbation, and show that grazing by these herbivores has prevented the establishment of macroalgae following near complete loss of coral on offshore reefs. Importantly, we found the positive response of herbivorous fishes to increased benthic primary productivity associated with coral loss was driven largely by parrotfishes that initially recruit to stable nursery habitat within the lagoons before moving to offshore reefs later in life. These results underscore the importance of connectivity between the lagoon and offshore reefs for preventing the establishment of macroalgae following disturbances, and indicate that protecting nearshore nursery habitat of herbivorous fishes is critical for maintaining reef resilience.

## Introduction

Understanding what controls the capacity of an ecosystem to return to its previous state following a perturbation and how human activities alter this capacity is centrally important for ecosystem based management [1]–[3]. Like many ecosystems, coral reefs have been subject to recurrent physical and biotic disturbances throughout their evolutionary history, and have demonstrated a capacity to consistently reassemble (i.e., return to coral dominance) following perturbations (e.g., [4]–[6]). By contrast, observations of coral reefs during recent decades reveal strikingly different dynamics with many reefs failing to return to coral dominance following major disturbances (e.g., [7]–[9]), a fate often attributed to a combination of human-induced drivers that have lowered the resilience of these systems [10]–[13]. Indeed, time-series data from some modern-day reefs indicate that in the absence of chronic local drivers, reefs can still recover from acute pulse disturbances on decadal time scales [14]–[16]. Given that the scale and the frequency of perturbations almost certainly will increase with global climate change [17], a top research priority is to identify the processes that cause some coral reef ecosystems to regain coral dominance following disturbances, while others persist in coral depauperate states [13].

Following large reductions in coral cover, many reefs—particularly those in the Caribbean—have become dominated by macroalgae [11], [13], [18]. Herbivory, therefore, has been identified as a key process influencing reef resilience, and overfishing of herbivorous fishes and collapse of herbivorous sea urchin populations have been implicated as underlying causes of algal dominance on many reefs in the Caribbean [7], [11], [19]. Nonetheless, our understanding of how the process of herbivory influences the capacity of reefs to recover from different disturbances is far from complete [11], a situation that hinders the development of effective management strategies for enhancing reef resilience. For example, we know little about what currently limits populations of herbivores on most reefs, and how these populations respond to large reductions in coral cover. In this study, we address these issues by exploring the dynamics of an Indo-Pacific coral reef that has undergone multiple cycles of perturbation over the past several decades without a switch to algal domination.

**Figure 1:**
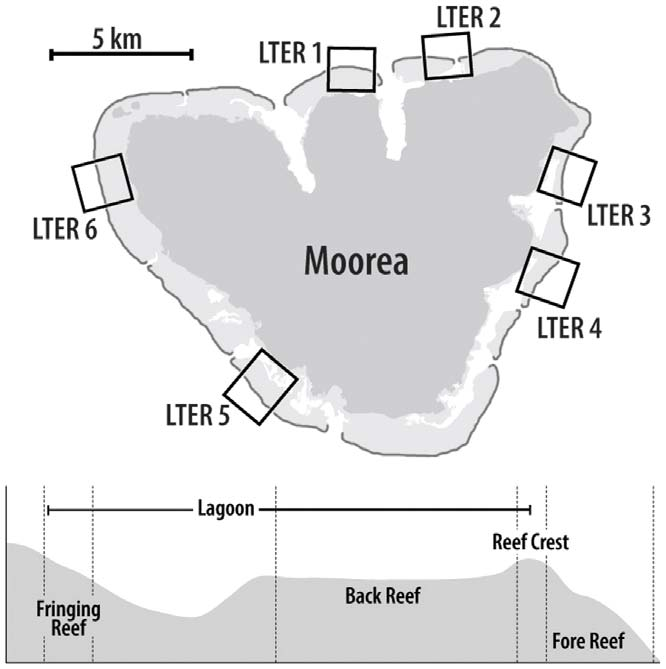
Map of Moorea showing different habitat types. Map of the island of Moorea with locations of sampling sites (LTER 1–LTER 6) and schematic illustrating the 3 habitat types sampled at each site. Habitat types are delineated with dotted lines; the fringing reef and backreef are located inshore of the reef crest and together make up the lagoon, while the forereef is located offshore of the reef crest. The predominant habitat types within the lagoons of Moorea are coral-based. Fringing reefs are often characterized by contiguous coral, while the backreef consists of a mosaic of small patch reefs separated by sand, rubble, and coral pavement.

Between 1980 and 2006, coral reefs surrounding Moorea, French Polynesia experienced several major perturbations, including an outbreak of corallivorous crown-of-thorns starfish (*Acanthaster planci*; hereafter COTS), multiple cyclones, and a number of bleaching events. These events disproportionately reduced coral cover on the forereef compared to the sheltered lagoon behind the reef crest [20], [21] (see Fig. 1). Cover of corals on the forereef was especially lowered by two large events, one in the early 1980's and another in the early 1990's [20], [21]. Following both of these events, the forereef community transitioned back to coral dominance on relatively short (decadal) time scales. Indeed, by 2006 coral cover on the forereef was at or near recorded highs [20], [21]. However, beginning in 2007, the forereef once again experienced a major perturbation in the form of a COTS outbreak, which caused mass mortality of coral on the forereef [22]. As in the two previous events, there was no shift to domination by macroalgae, suggesting that herbivores were able to control the growth of macroalgae or that conditions on the forereef were not conducive to algal growth. Here we use time-series data, behavioral observations, and a field experiment to identify the processes that prevented a shift towards macroalgal dominance and thereby contribute to the resilience of the ecosystem. Our findings have important implications for development of ecosystem-based management strategies to enhance resilience of coral reefs.

## Results

**Figure 2:**
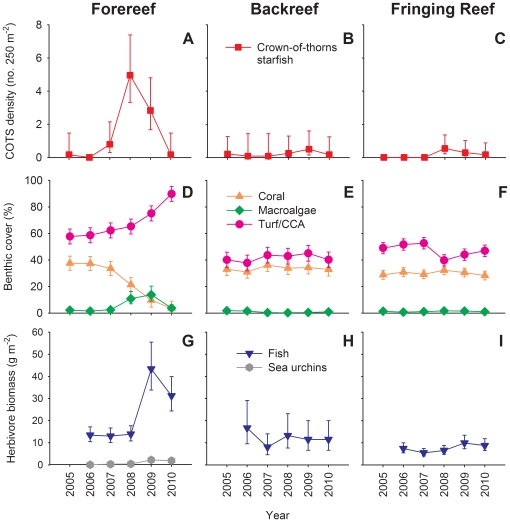
Dynamics of reef organisms. Dynamics (mean ± 95% CI) of (A–C) corallivorous COTS, (D–F) coral and algae, and (G–I) herbivore biomass on the forereef, backreef, and fringing reef habitats at six sites on Moorea. The key for each row of panels is located in the middle panel. Biomass estimates for herbivorous sea urchins on the backreef and fringing reef habitats are not available; abundance data are presented in Fig. S1.

Time-series data from six sites distributed around Moorea revealed significant variation among years in the density of COTS on the forereef, with COTS beginning to increase in density in 2007, reaching peak densities in 2008 and 2009, and then abruptly declining in 2010 (GLM P<0.0001; Fig. 2). The cover of live coral on the forereef declined by ∼90%, from ∼40% in 2005 to <5% in 2010 (Fig. 2; Table S1). Concurrent with the decline in coral cover was an increase in reef substrate suitable for algal growth followed by a modest but significant increase in macroalgae (largely *Halimeda* spp.) in 2008 and 2009 (Fig. 2; Table S1). By 2010, macroalgae had returned to pre-disturbance levels, and ∼90% of the substrate on the forereef was occupied by closely cropped filamentous turfing algae and/or crustose coralline algae (Fig. 2; Table S1). Between 2008 and 2010, roving herbivorous fishes on the forereef nearly doubled in density and tripled in total biomass (Fig. 2; Table S2), while herbivorous sea urchins increased in density more than 4-fold (Fig. S1; Table S3). However, in 2010 sea urchins still accounted for only a very small portion (<5%) of total herbivore biomass on the forereef (Fig. 2). Temporal patterns of abundance in the lagoons differed sharply from those on the forereef. Throughout the study, COTS were an order of magnitude less abundant on the backreef and fringing reef compared to the peak densities observed on the forereef (Fig. 2). In addition, there were no consistent temporal trends in coral cover on the backreef or fringing reef, nor were there any concomitant changes in the abundance or biomass of herbivorous fishes or sea urchins in either habitat (Fig. 2, Fig. S1; Tables S1, S2, and S3).

**Figure 3:**
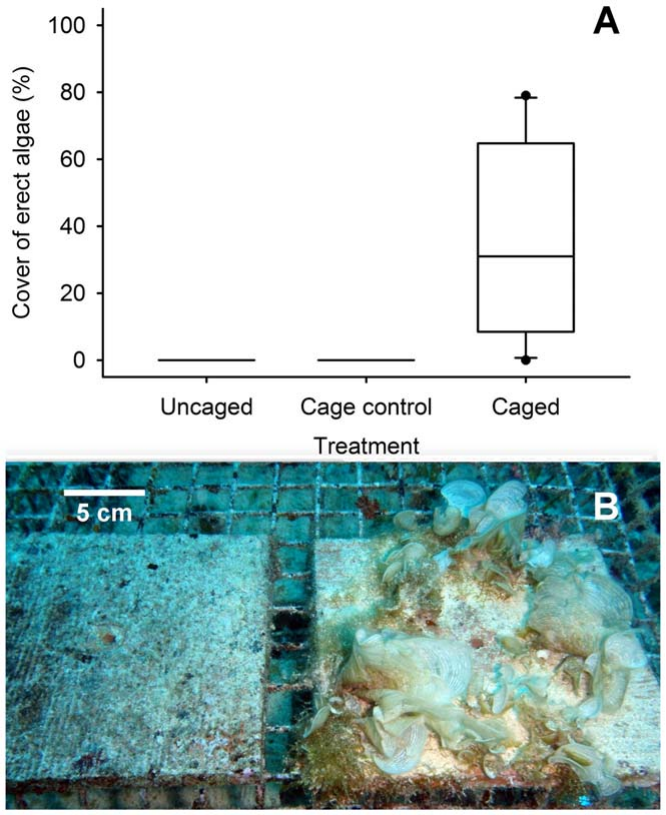
Results of herbivore exclusion experiment. (A) Box and whisker plot of percent cover of erect algae on tiles exposed (Uncaged and Cage control) and unexposed (Caged) to ambient grazing by herbivorous fishes (n = 10). Erect algae were predominately macroalgae (63%), but also include mats of articulated coralline algae (26%) and the cyanobacteria *Symploca hydnoides* (11%). Boxes are medians with 25^th^ and 75^th^ quartiles. Whiskers are the 10^th^ and 90^th^ percentiles, and dots show the range of the data. (B) Tiles from a cage control (left) and a cage (right, covered with the macroalgae *Padina boryana*) from the same experimental block.

To determine whether herbivorous fishes were necessary to prevent the establishment of macroalgae, we conducted a field experiment on the forereef where large fishes were allowed to, or prevented from, gaining access to standardized substrate (terra cotta tiles) for 16 weeks. During this time, most tiles protected from large fishes were colonized by macroalgae (mainly *Padina boryana* and *Sargassum pacificum*) with cover ranging from 0 to 72% (median = 14.5%) (Fig. 3). Several protected tiles also were colonized by tufts of the cyanobacteria *Symploca hydnoides* and thick mats of articulated coralline algae, which together with macroalgae dominated the biomass of most caged tiles. Macroalgae, *Symploca hydnoides*, and thick mats of articulated coralline algae were absent from tiles exposed to ambient grazing (uncaged and cage controls), which were dominated by very closely cropped filamentous turfing algae (Fig. 3).

**Figure 4:**
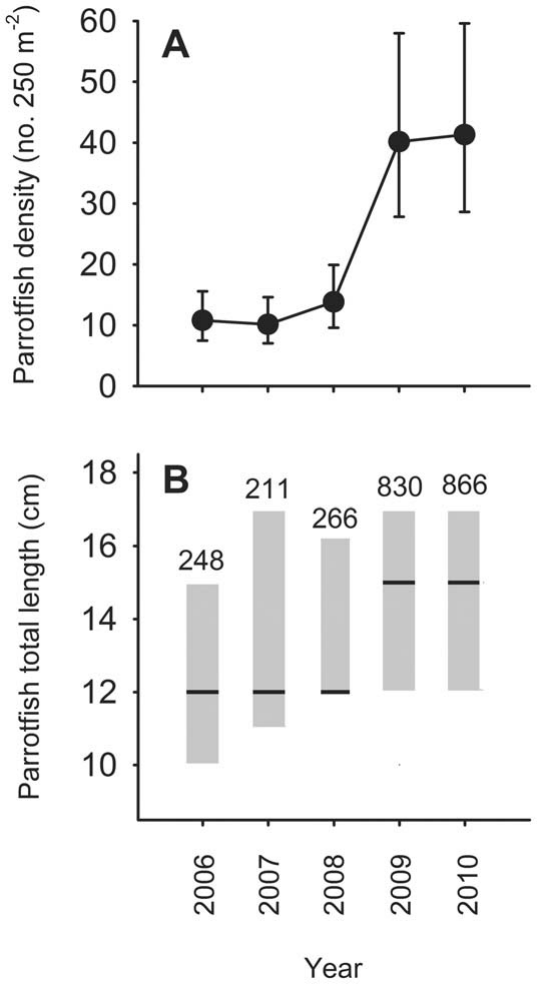
Parrotfish dynamics. (A) Temporal pattern of abundance (mean±95% CI) of parrotfish on the forereef. (B) Box plot showing changes in the median size of parrotfish on the forereef. Lines are the median size and boxes encompass the 25^th^ and 75^th^ percentiles. Sample sizes are given above each box.

**Figure 5:**
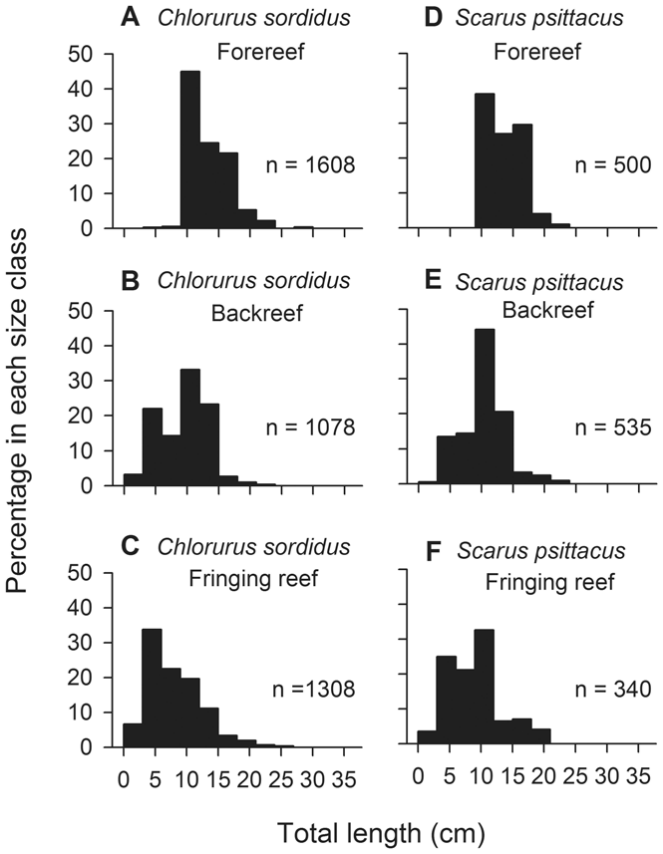
Ontogenetic patterns of habitat use for the two most abundant species of parrotfish. Size frequency distributions for (A–C) *Chlorurus sordidus* and (D–F) *Scarus psittacus* on the forereef, backreef, and fringing reef. Data are pooled from all sites and years.

**Figure 6:**
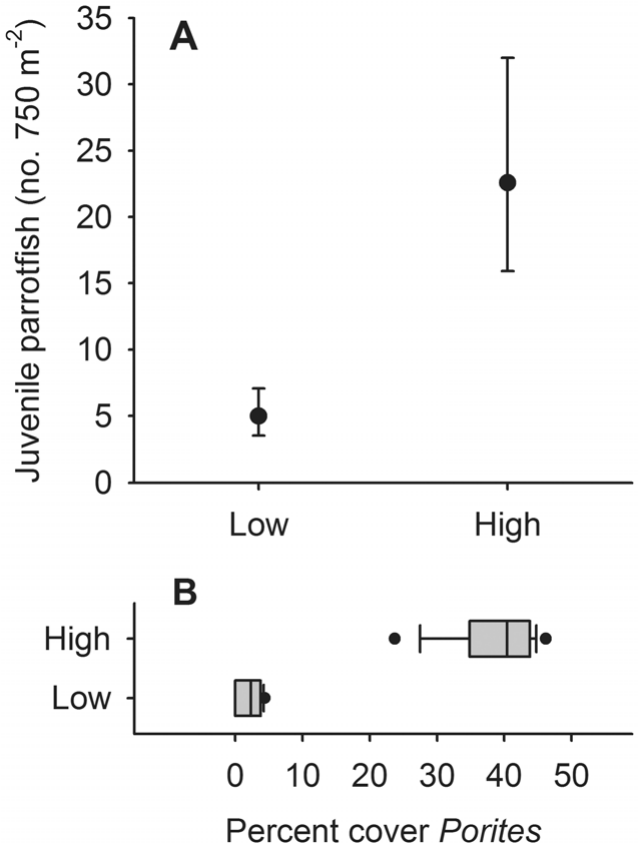
Association between juvenile parrotfish and the coral *Porites rus*. (A) Mean (±95% CI) number of juvenile parrotfish (*C. sordidus* and *S. psittacus*) observed at fringing reefs with high (n = 3) and low (n = 3) cover of the coral *Porites rus*. Analyses were conducted on log transformed data; means and error bars are back-transformed. (B) Box and whisker plot showing the distribution of coral cover values at the three sites with high and low levels of *Porites rus* for each of the five survey years. Boxes are medians with 25^th^ and 75^th^ quartiles. Whiskers are the 10^th^ and 90^th^ percentiles, and dots show the range of the data.

Following the decline in cover of live coral, the herbivore assemblage on the forereef became increasingly dominated by parrotfish. For example, in 2006 parrotfish accounted for ∼22% of the biomass of roving herbivorous fishes on the forereef; however, by 2010 parrotfish had increased relative to other herbivores and accounted for ∼50% of the biomass. Parrotfish biomass in turn was dominated by two species, *Chlorurus sordidus* and *Scarus psittacus*, which together accounted for ∼80% of the total parrotfish biomass on the forereef in 2010. Between 2008 and 2010, parrotfish on the forereef increased in abundance approximately 4-fold (Fig. 4A, Table S4, also see Figs. S2 and S3 for the dynamics of individual species). In addition, their median length increased from 12 cm in 2006–2008 to 15 cm in 2009–2010 (Kruskal Wallis, P<0.0001; Fig. 4B). Throughout this period, small juvenile parrotfish were virtually absent from the forereef, and inspection of size frequency distributions strongly suggest they initially recruit to the fringing reef and backreef and then move to the forereef after reaching a length of ∼10 cm (Fig. 5; see also Figs. S4 and S5). Targeted surveys of juvenile parrotfish revealed that 92% of small (<5 cm TL) juvenile *C. sordidus* and *S. psittacus* were associated with the live coral *Porites rus* (471 of 514 individuals and 107 of 115 groups encountered), which is abundant in many of the lagoon habitats of Moorea, but uncommon on the forereef. Finally, small juvenile *C. sordidus* and *S. psittacus* were approximately five times as abundant at fringing reef sites dominated by *P. rus* than at sites with little *P. rus* (ANOVA, F_1,4_ = 72.19, P = 0.0011; Fig. 6).

## Discussion

Coral reefs worldwide are experiencing unprecedented threats from a combination of local drivers, such as overfishing and pollution, and global drivers associated with climate change [13]. While some reefs have exhibited the capacity to return to coral dominance following large-scale perturbations (e.g., [15], [16], [23]), others have failed to do so, and many of these have become dominated by macroalgae (e.g., [24]–[26]). Because shifts to macroalgal dominance appear to be easier to prevent than reverse [11], processes that occur shortly after coral decline likely are critical for preventing persistent state shifts to macroalgae. Our findings indicate that after near complete loss of coral, the forereef community on Moorea has not become dominated by macroalgae. Rather, coral decline was accompanied by a rapid and sustained increase in populations of herbivorous fish (particularly parrotfish) whose intense grazing has kept algae in a closely cropped state. Both the magnitude and rapidity of this response were striking, in part because many coral reef fishes are predicted to decline in abundance in response to reductions in coral cover [27]–[29], but also because it had been suggested that herbivorous fish in Moorea might be limited by moderate levels of fishing [30]. However, our results strongly suggest that herbivorous fish were food limited prior to the COTS outbreak and that an increase in benthic primary production associated with coral decline stimulated rapid population growth.

While populations of coral reef fishes are frequently limited by the availability of juvenile habitat (e.g., [31]), and loss of coral can lead to widespread recruitment failure of fishes by eliminating this critical habitat [32], [33], we found that parrotfish in Moorea primarily settle into the lagoon, where the COTS outbreak had little impact on coral cover, and then move to the forereef later in life when they are no longer closely associated with coral habitat. Consequently, parrotfish populations were able to respond to the increased food availability on the forereef in part because their juvenile habitat was unaffected by the COTS outbreak. These results strongly support the idea that connectivity between offshore reefs and inshore nursery habitats can enhance the resilience of coral reefs, and indicate that protecting nursery habitats—including mangroves, seagrasses, and inshore reefs—should be a top management priority [34]–[38].

It is widely recognized that grazing is a key ecosystem process on coral reefs, yet the mechanisms by which different disturbances affect grazing levels are not well understood. For example, while it is clear that grazing has been important in facilitating the return to coral dominance of some reefs following large disturbances, it is less clear whether rapid behavioral responses of herbivores (including increased feeding rates and the redistribution of individuals from less productive habitats) or population growth have been responsible for absorbing the increased primary production associated with coral decline [39], [40]. Importantly, our results demonstrate that increases in the biomass of herbivorous fishes on the forereef of Moorea were due to population growth and increases in biomass of individuals, and not simply redistribution among habitats. Indeed, the two most abundant species of parrotfish, *C. sordidus* and *S. psittacus* did not decline in the lagoon, suggesting increased settlement and/or survivorship of individuals within the lagoon over the study period. While behavioral responses of herbivores undoubtedly play a key role in preventing shifts to macroalgal dominance immediately following decline in live coral, our results indicate that these can be accompanied by rapid population increases that could be equally important for preventing the establishment of macroalgae over somewhat longer time scales. Indeed, increases in abundance of herbivores have been observed following coral decline on other reefs [27], and a recent synthesis suggests that populations of herbivorous fish may be at lower risk from climate-driven disturbances than most other reef fishes [29]. Our results illustrate that responses will be contingent on the availability of nursery habitats as well as the ability of fishes to move between these habitats and offshore coral reefs.

Inshore nursery habitats worldwide, including seagrasses and mangroves, as well as spatially and taxonomically distinct inshore reefs, commonly harbor important herbivorous fishes (especially parrotfishes) that later migrate to offshore coral reefs [34], [41]–[43]. Connectivity between inshore nurseries and offshore coral reefs could contribute greatly to resilience if the dynamics of juvenile habitats for herbivorous fishes are decoupled from those of offshore reefs. Indeed, habitat-providing species in inshore nurseries are likely to respond differently to perturbations—including those associated with climate change—than corals on offshore reefs [44]. For example, our results indicate that corals in inshore nurseries in Moorea underwent strikingly different dynamics in response to the COTS outbreak than corals on nearby offshore reefs, with coral cover in the lagoon changing little despite near complete loss of coral along ∼50 km of coastline on the forereef. Additionally, these dynamics are representative of longer term patterns; coral cover on Moorea has been much less variable in the lagoon than on the forereef over the past three decades despite a number of major disturbances (including cyclones, COTS outbreaks, and bleaching events) [21]. Furthermore, because many lagoon reefs in Moorea are dominated by massive corals in the genus *Porites*, which are resistant to coral bleaching [45], [46], and may be well suited for acclimatization to changing conditions in general [47], these reefs could be more resistant to climate-driven perturbations in the future [48]–[50].

An increasing number of disturbances to coral reefs are driven by global phenomena (i.e., widespread bleaching in response to rising sea surface temperatures), and hence, there have been calls for local management efforts to focus on conserving processes that enhance the ability of these ecosystems to return to coral dominance following perturbations (i.e., managing for resilience) [11], [13], [51]. The most commonly discussed resilience-based management strategy for coral reefs focuses largely on limiting exploitation of functionally important species such as herbivorous fishes (e.g. [11], [13], [29]). While fisheries management will be a critical component of any ecosystem based management strategy, our results also highlight the importance of juvenile habitat for herbivorous fishes, and we would caution against over-simplified management strategies that focus solely on fishing while failing to explicitly consider the degradation of nearshore habitats. Importantly, many nursery habitats used by herbivorous fishes, including mangroves, seagrasses, and some inshore coral reefs, appear to be threatened to a greater extent by coastal development than climate change [52]–[54], and hence, these habitats could benefit greatly from local management action. While networks of marine reserves are increasingly being established as a fishery management strategy [51], [55], few networks aimed at protecting coral reef ecosystems explicitly consider connectivity with nearby nursery habitats [36]. Furthermore, appropriate management strategies for coral reefs and nearshore nurseries will require considerations beyond fisheries management, including impacts originating from the terrestrial environment, especially eutrophication and sedimentation [56]. For example, nearshore nursery areas in Moorea—while likely to be more resistant to climate change associated disturbances than offshore reefs—are also at the highest risk from land-based pollution which cannot be managed solely by the establishment of marine reserves. Recognition of the many linkages that exist across ecosystem boundaries provides a broader perspective of connectivity on coral reefs that will contribute to the development of more effective local management strategies in the face of global climate change.

## Materials and Methods

### Time series data collection and analysis

All research was performed under annual research permits issued by the French Polynesian Ministry of Research to the Moorea Coral Reef LTER, and in accordance with University of California Santa Barbara's Institutional Animal Care and Use (IACUC) Protocol # 639. The Moorea Coral Reef Long-Term Ecological Research site (MCR LTER) has collected time series data annually in three habitat types (the forereef, backreef, and fringing reef,) at six sites around the island of Moorea, French Polynesia since 2005 (see Fig. 1). Fixed transects were established at each site using a stratified random design, and data on benthic cover, mobile invertebrates, and fishes are collected by SCUBA divers. On the forereef, benthic cover and mobile invertebrates are sampled at two depths (10 m and 17 m), while fishes are sampled at a single depth (∼12 m); analyses of benthic data presented here are from the 10 m depth which is directly adjacent to the fish transects. At each site-habitat-depth combination, benthic cover is assessed in fixed 0.5 m×0.5 m quadrats located randomly along five 10 m transects (n = 40). Quadrats are photographed and the cover of the major benthic components (i.e., scleractinian corals (usually to genus), macroalgae, turf algae) quantified using 200 random point contacts per quadrat (generated with CPCe software [57]). Mobile invertebrates are counted in fixed 1 m×1 m quadrats located randomly along five 10 m transects (n = 20), and fish and crown-of-thorns starfish (COTS, *Acanthaster planci*) are counted on four 50 m transects. Fish transects extend from the sea floor to the surface of the water column and consist of two swaths surveyed sequentially. Divers first count mobile fish on a 5 m wide swath before counting cryptic benthic fishes on a 1 m wide swath; total lengths (TL) of fish are estimated to 0.5 cm. Additional details on sampling protocols can be viewed at: http://mcr.lternet.edu/data/.

To test for island-wide changes in the densities of COTS in each of the three habitat types, we used generalized linear models with a quasipoisson distribution (to account for overdispersion) and log link function. Changes in the percent cover of coral and algae and in the density and biomass of herbivorous fishes and sea urchins were evaluated using mixed-effects ANOVA (fixed effect = year, random effect = site). Fishes were categorized as herbivorous if they fed primarily on algae (filamentous or fleshy) and/or detritus (mainly surgeonfishes and parrotfishes). Biomass of herbivorous fishes was estimated using published length/weight relationships [58]. In contrast to fish, the body sizes of sea urchins are not estimated in our surveys. To compare the biomass of herbivorous sea urchins and fish on the forereef, the biomass of each sea urchin species was estimated using representative size distributions from forereef populations in Moorea and published length-weight relationships. For both fish and sea urchins we focused on species likely to be important in controlling the establishment and growth of macroalgae. As such, the sea urchin *Echinostrephus aciculatus*, which feeds primarily on drift algae, was excluded from calculations of herbivore abundance and biomass, as were small, territorial herbivorous fishes (mainly small damselfishes, angelfishes and blennies). Additional methodological details are presented in Text S1.

### Experiment to assess whether herbivory by fishes controls the establishment of macroalgae

To determine whether conditions were amenable for the establishment of macroalgae on the forereef, we compared the communities that developed after ∼16 wks when large herbivorous fish were experimentally excluded with those that developed under ambient grazing. To accomplish this, we established ten replicates of three treatments (caged to exclude herbivorous fish, uncaged to allow access, and cage control) in a randomized block design at a depth of ∼12 m on the north shore of Moorea. We then measured percent cover (from 100 uniform point contacts) and biomass (ash-free dry weight) of algae (and cyanobacteria) accumulating on 15 cm×15 cm terra cotta tiles exposed (uncaged and cage control treatments) and unexposed (caged treatment) to grazing by herbivorous fishes. Cages had a mesh size of 2.5 cm×2.5 cm which was small enough to exclude herbivorous fishes (but not sea urchins) but large enough to minimize cage artifacts. Cage controls were identical to full cages with the exception that they were missing two opposing sides and the top to allow access by herbivorous fish. Terra cotta tiles were “seasoned” in the lagoon for several months prior to use in the experiment which was initiated on July 28, 2010.

### Response of herbivorous fish to reduction in coral cover

The increase in biomass of herbivorous fish observed on the forereef between 2008 and 2010 was driven largely by parrotfish. To explore further the processes influencing parrotfish dynamics, we investigated changes in their density and size structure on the forereef. In addition, differences in the size structure of parrotfish among habitats indicated an ontogenetic shift in habitat use from the lagoon (backreef and fringing reef) to the forereef; consequently, we tested whether there were differences in median body size among the three habitat types. Finally, to quantify habitat associations of juvenile parrotfish, we searched for juveniles and noted their microhabitat (substrate) associations on thirty-four 50 m×1 m transects, encompassing the full range of habitats (to 16 m depth) found in the lagoon and forereef, as well as eight 100 m×10 m transects at two fringing reef and two backreef sites; surveys were conducted during the 2010 Austral winter and 2011 Austral summer. Additionally, because these surveys revealed an association between small (<5 cm TL) juvenile *C. sordidus* and *S. psittacus* and the coral *Porites rus*, time series data were used to test whether fringing reef sites dominated by *P. rus* harbored more small juveniles than sites with little *P. rus*. Temporal changes in the density of parrotfish were evaluated with mixed-effects ANOVA (fixed effect = year, random effect = site); data were pooled at the site level and log transformed to improve distributional properties. Differences in median body size were evaluated with Kruskal-Wallis tests followed by adjusted pairwise comparisons with data pooled across sites and years. Finally, we used ANOVA to test whether there were more small juvenile *C. sordidus* and *S. psittacus* at fringing reef sites dominated by *P. rus* than at sites with little *P. rus*; data were averaged across years and log transformed.

## Supporting Information

Text S1 **Detailed methods and results.**
(DOC)

Figure S1 **Dynamics of herbivorous sea urchins.** Patterns of abundance (mean±95%) of herbivorous sea urchins on the (A) forereef, (B) backreef, and (C) fringing reef.
(TIF)

Figure S2 **Size frequency distributions of** ***C. sordidus*** **in each of the three habitat types over time.** Between 2008 and 2010 *C. sordidus* doubled in density on the forereef while shifting in median length from 12 to 15 cm, together resulting in a tripling in biomass. Size distributions differed among habitats with nearshore habitats having a greater proportion of small individuals.
(TIF)

Figure S3 **Size frequency distributions of** ***S. psittacus*** **in each of the three habitat types over time.** Between 2008 and 2010 *S. psittacus* increased in density and biomass more than 20-fold. Size distributions differed among habitats with nearshore habitats having a greater proportion of small individuals.
(TIF)

Figure S4 **Size frequency distributions of** ***C. sordidus*** **surveyed twice annually at 13 sites between 2004 and 2008.** Distributions show consistent ontogenetic patterns of habitat use among seasons.
(TIF)

Figure S5 **Size frequency distributions of** ***S. psittacus*** **surveyed twice annually at 13 sites between 2004 and 2008.** Distributions show consistent ontogenetic patterns of habitat use among seasons.
(TIF)

Table S1 **Results of mixed effects ANOVA on the cover of (a) coral, (b) macroalgae, and (c) bare space/turf/CCA in each of the three habitat types.** Results of post hoc Tukey tests for the fixed effect of year are indicated; years not sharing the same letter are significantly different at P<0.05.
(DOC)

Table S2 **Results of mixed-effects ANOVA on the (a) density and (b) biomass of herbivorous fish in each of the three habitat types.** Results of post hoc Tukey tests for the fixed effect of year are indicated; years not sharing the same letter are significantly different at P<0.05.
(DOC)

Table S3 **Results of mixed-effects ANOVA on the density of herbivorous sea urchins in each of the three habitat types.** Results of post hoc Tukey tests for the fixed effect of year are shown; years not sharing the same letter are significantly different at P<0.05.
(DOC)

Table S4 **Results of mixed-effects ANOVA on the density of parrotfishes in each of the three habitat types.** Results of post hoc Tukey tests for the fixed effect of year are shown; years not sharing the same letter are significantly different at P<0.05.
(DOC)
