# Supplementary material for: Herbivory, Connectivity, and Ecosystem Resilience: Response of a Coral Reef to a Large-Scale Perturbation
Source: PLoS One. 2011 Aug 25;6(8):e23717. doi: 10.1371/journal.pone.0023717 (PMC3162008; doi:10.1371/journal.pone.0023717)
Supplement: Text S1 — Detailed methods and results. (DOC) [file pone.0023717.s001.doc]

**Text S1 Detailed methods and results**

***Changes in the density of COTS: analyses and results***

To test for changes in the density of COTS through time in each of the three habitat types, we used generalized linear models with a quasipoisson distribution (to account for overdispersion) and log link function. Data from individual transects were pooled at the site level. Variation in the abundance of COTS among years was significant on the forereef (GLM, *P* < 0.0001) as well as the fringing reef (GLM, *P* = 0.01248), but not the backreef (GLM, *P* = 0.6845). However, densities on the fringing reef and backreef were low throughout the time period and were consistently at least an order of magnitude lower than the peak densities observed on the forereef.

***Changes in benthic cover: analyses and results***

To test for changes in percent cover of coral and macroalgae in each of the three habitat types, we used mixed effects ANOVA followed by post-hoc Tukey tests. Mean percent cover from all quadrats at each site were used in analyses. Cover of macroalgae was logit [ln(x/(1-x))] transformed to improve distributional properties. Each model included year as a fixed effect and site as a random effect. Results are presented in Table S1.

***Changes in the density and biomass of herbivorous fishes: analyses and results***

To test for changes in the density and biomass of herbivorous fishes through time in each of the three habitat types, we used mixed-effects ANOVA, followed by post-hoc Tukey tests. Each model included year as a fixed effect and site as a random effect. For the analyses of biomass, data from individual transects were pooled at the site level to improve distributional properties. By contrast, density data were not pooled and models included the additional random effect of transect nested in the site effect. All analyses were conducted on log transformed data. Results are presented in Table S2.

***Changes in the density of herbivorous sea urchins: analyses and results***

To test for changes in the density of herbivorous sea urchins through time in each of the three habitat types, we used mixed-effects ANOVA, with year as a fixed effect and site as a random effect followed by post-hoc Tukey tests. Data were log transformed and pooled at the site level to improve distributional properties. Results are presented in Table S3 and Figure S1

***Size structure and dynamics of parrotfishes***

Between 2008 and 2010, parrotfish on the forereef increased in abundance approximately 4-fold (Table S4). Parrotfish biomass on the forereef was dominated by two species, *Chlorurus sordidus* and *Scarus psittacus*. Together, these two species accounted for ~ 80% of the total parrotfish biomass on the forereef in 2010. Both increased in density on the forereef between 2008 and 2010 with *C. sordidus* approximately doubling in density, and *S. psittacus* increasing more than 20-fold (Figs. S2 and S3). *C. sordidus* also experienced a shift in the size distribution of the population with larger individuals occurring on the forereef in 2009 and 2010 than in years prior (Fig. S2). Finally, for both species, individuals less than 10 cm TL were common on the backreef and fringing reef and virtually absent from the forereef during all years (Figs. S2 and S3).

While differences in the size structure of parrotfish among habitats strongly suggested an ontogenetic shift in habitat use from the lagoon (backreef and fringing reef) to the forereef, an alternative hypothesis is that parrotfish settle directly to the forereef and then grow out of the smallest size classes before they are counted in our annual surveys. To address this hypothesis we compared size frequency distributions of parrotfish surveyed at ~ 6 month intervals (Figs. S4 and S5). These data were collected by the Moorea PGEM Consortium to evaluate the impact of marine protected areas at 13 sites around the island of Moorea (see [1]). Data presented here are from surveys conducted between 2004 and 2008. For surveys that are conducted during the Austral winter, size frequency distributions of *C. sordidus* and *S psittacus* are consistent with the MCR LTER time series data. Furthermore, data collected during the Austral summer exhibit the same qualitative pattern, indicating that small (< 10 cm) parrotfish are absent from the forereef throughout the year.

***Estimating the biomass of herbivorous sea urchins***

Since the sizes of sea urchins were not measured during collection of the time series data, we estimated the biomass of sea urchins using size distributions from representative forereef populations. To accomplish this, in July/August 2010 test diameters of all sea urchins in thirty 5 m X 5 m plots at two representative forereef sites (one N shore site (n = 20 plots) and one E shore site (n = 10 plots)) were measured, and distributions of weights for each species were calculated using published length-weight relationships [2, 3]. Biomass estimates from the time series data on sea urchin abundance were generated by sampling at random (with replacement) from these distributions to obtain urchin sizes. In 2010 the estimated biomass of herbivorous sea urchins on the forereef was 1.89 g m^-2^ (bootstrap confidence intervals: 1.32 – 2.58 g m^-2^), accounting for approximately 5% of the total biomass of macroherbivores. If the maximum size encountered for each species is used in the calculation the upper limit of estimated sea urchin biomass on the forereef in 2010 would be 9.53 g m^-2^, which would account for approximately 20% of the total herbivore biomass in that year.

References

1. Lison de Loma T, Osenberg CW, Shima JS, Chancerelle Y, Davies N, Brooks AJ, Galzin R (2008) A framework for assessing impacts of marine protected areas in Moorea (French Polynesia). *Pacific Science* 62:431-441.

2. Muthiga NA, McClanahan TR (1987) Population changes of a sea urchin (*Echinometra mathaei*) on an exploited fringing reef. *Afr J Ecol* 25:1-8.

3. McClanahan TR (1988) Coexistence in a sea urchin guild and its implications to coral reef diversity and degradation. *Oecologia* 77:210-218.

Figure Legends

**Figure S1. Dynamics of herbivorous sea urchins.** Patterns of abundance (mean + 95%) of herbivorous sea urchins on the (A) forereef, (B) backreef, and (C) fringing reef.

**Figure S2. Size frequency distributions of *C. sordidus*, in each of the three habitat types over time.** Between 2008 and 2010 *C. sordidus* doubled in density on the forereef while shifting in median length from 12 to 15 cm, together resulting in a tripling in biomass. Size distributions differed among habitats with nearshore habitats having a greater proportion of small individuals.

**Figure S3. Size frequency distributions of *S. psittacus* in each of the three habitat types over time.** Between 2008 and 2010 *S. psittacus* increased in density and biomass more than 20-fold. Size distributions differed among habitats with nearshore habitats having a greater proportion of small individuals.

**Figure S4. Size frequency distributions of *C. sordidus* surveyed twice annually at 13 sites between 2004 and 2008.**  Distributions show consistent ontogenetic patterns of habitat use among seasons.

**Figure S5. Size frequency distributions of *S. psittacus* surveyed twice annually at 13 sites between 2004 and 2008**. Distributions show consistent ontogenetic patterns of habitat use among seasons.
