# Supplementary figures and images for: Herbivory, Connectivity, and Ecosystem Resilience: Response of a Coral Reef to a Large-Scale Perturbation
Source: PLoS One. 2011 Aug 25;6(8):e23717. doi: 10.1371/journal.pone.0023717 (PMC3162008; doi:10.1371/journal.pone.0023717)

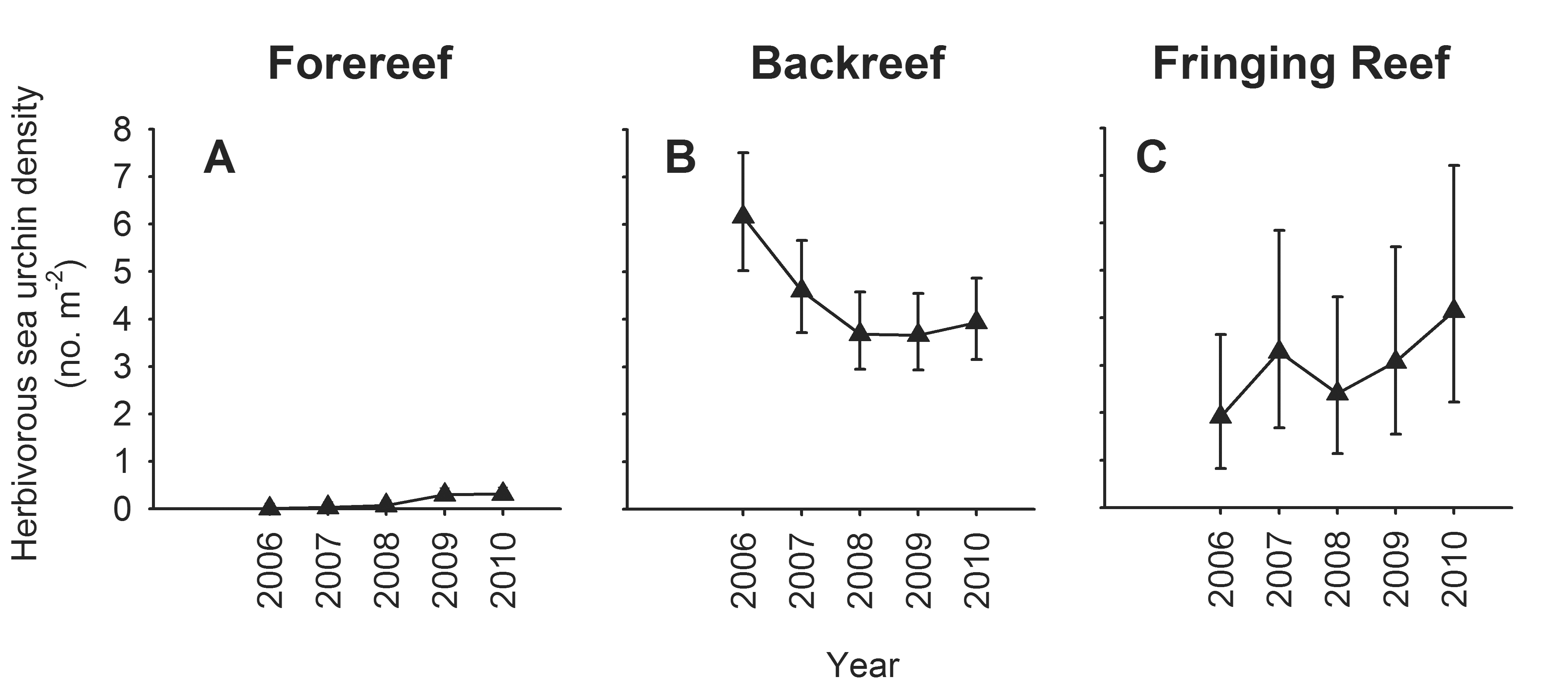

Supplement: Figure S1 — Dynamics of herbivorous sea urchins. Patterns of abundance (mean±95%) of herbivorous sea urchins on the (A) forereef, (B) backreef, and (C) fringing reef. (TIF) [file pone.0023717.s002.tif]

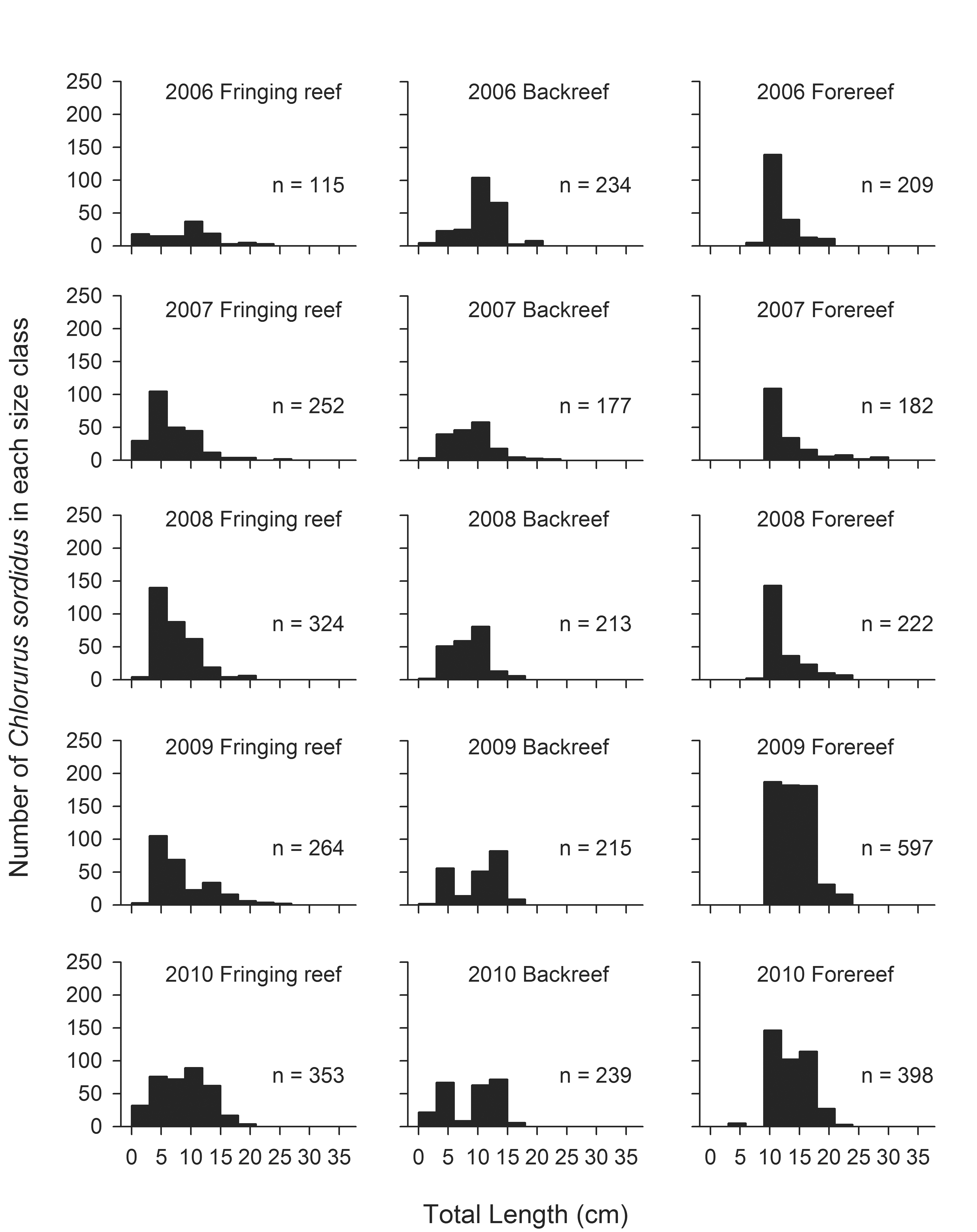

Supplement: Figure S2 — Size frequency distributions of C. sordidus in each of the three habitat types over time. Between 2008 and 2010 C. sordidus doubled in density on the forereef while shifting in median length from 12 to 15 cm, together resulting in a tripling in biomass. Size distributions differed among habitats with nearshore habitats having a greater proportion of small individuals. (TIF) [file pone.0023717.s003.tif]

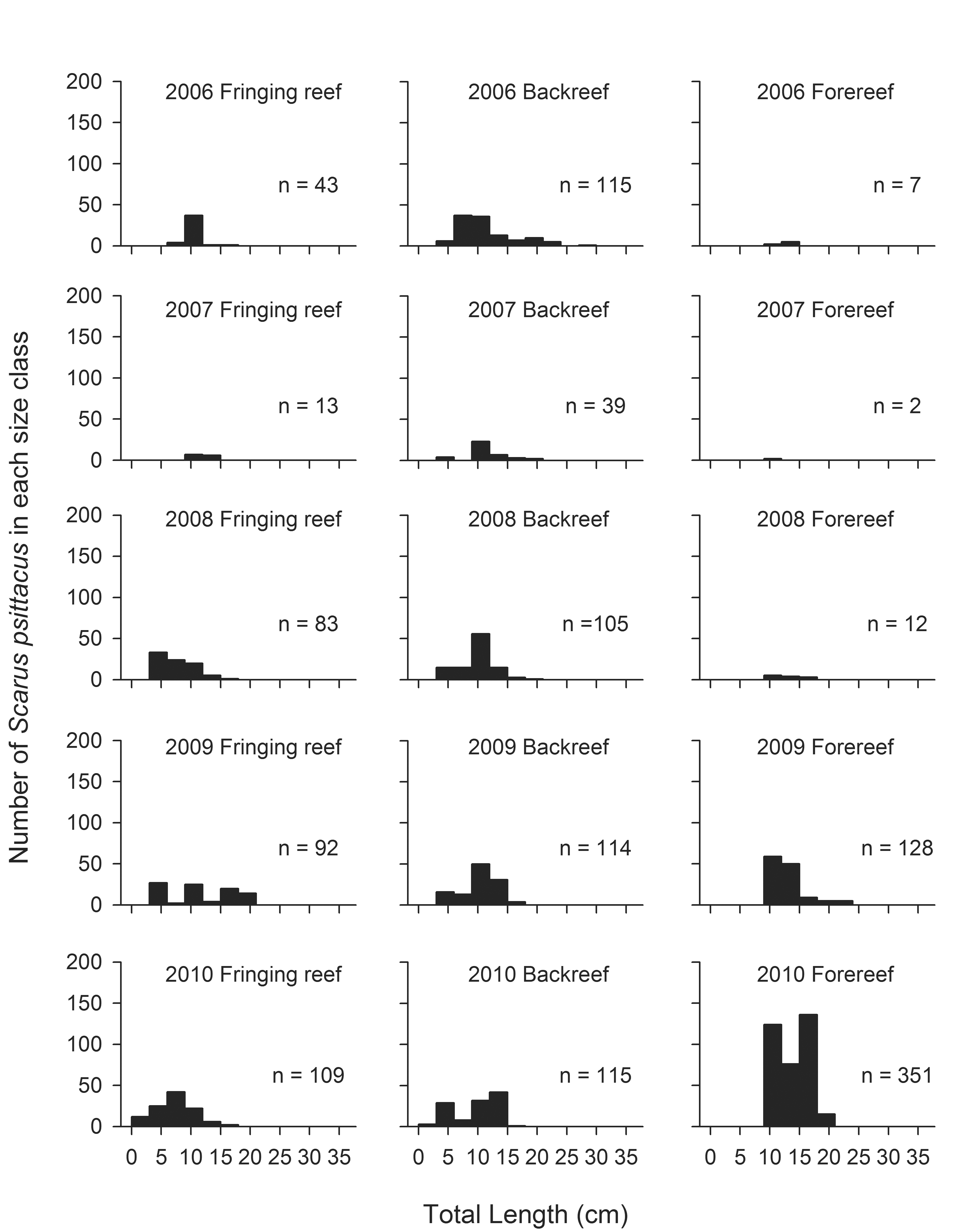

Supplement: Figure S3 — Size frequency distributions of S. psittacus in each of the three habitat types over time. Between 2008 and 2010 S. psittacus increased in density and biomass more than 20-fold. Size distributions differed among habitats with nearshore habitats having a greater proportion of small individuals. (TIF) [file pone.0023717.s004.tif]

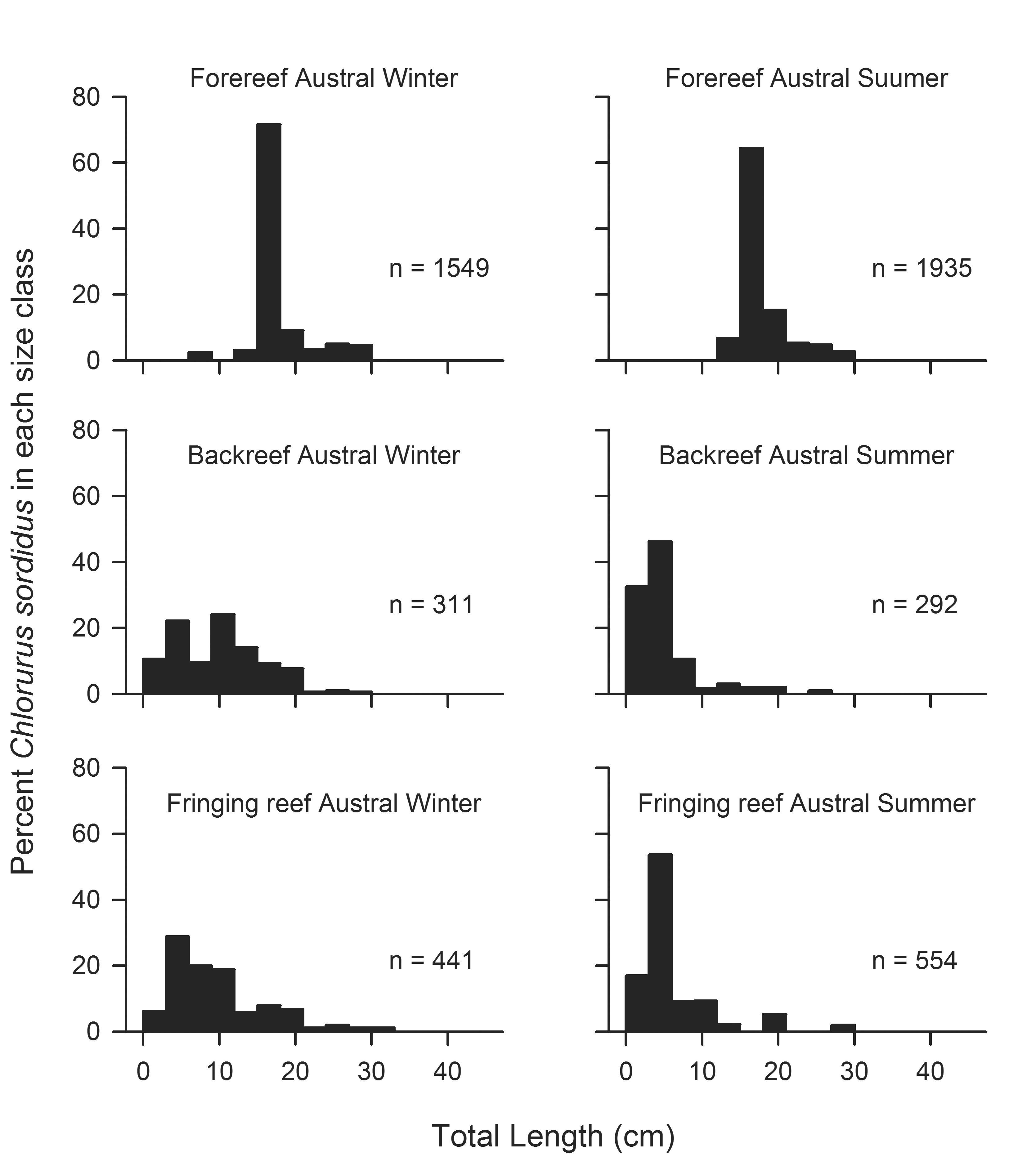

Supplement: Figure S4 — Size frequency distributions of C. sordidus surveyed twice annually at 13 sites between 2004 and 2008. Distributions show consistent ontogenetic patterns of habitat use among seasons. (TIF) [file pone.0023717.s005.tif]

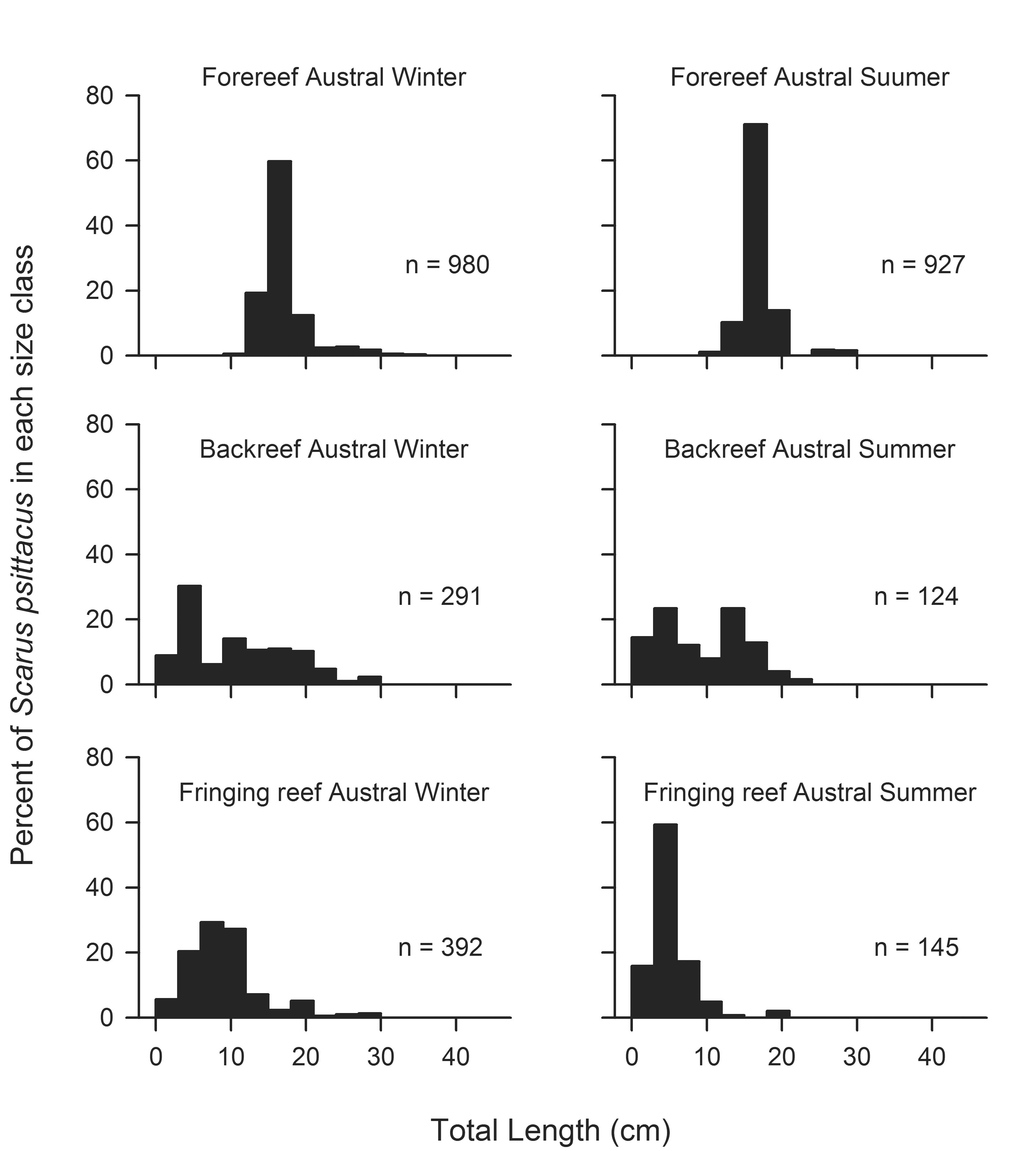

Supplement: Figure S5 — Size frequency distributions of S. psittacus surveyed twice annually at 13 sites between 2004 and 2008. Distributions show consistent ontogenetic patterns of habitat use among seasons. (TIF) [file pone.0023717.s006.tif]
