# Supplementary material for: Herbivory, Connectivity, and Ecosystem Resilience: Response of a Coral Reef to a Large-Scale Perturbation
Source: PLoS One. 2011 Aug 25;6(8):e23717. doi: 10.1371/journal.pone.0023717 (PMC3162008; doi:10.1371/journal.pone.0023717)
Supplement: Table S1 — Results of mixed effects ANOVA on the cover of (a) coral, (b) macroalgae, and (c) bare space/turf/CCA in each of the three habitat types. Results of post hoc Tukey tests for the fixed effect of year are indicated; years not sharing the same letter are significantly different at P<0.05. (DOC) [file pone.0023717.s007.doc]

Table S1. Result of mixed effects ANOVA on the cover of (a) coral, (b) macroalgae, and (c) bare space/turf/CCA in each of the three habitat types. Results of post hoc Tukey tests for the fixed effect of year are indicated; years not sharing the same letter are significantly different at P < 0.05.

| Source of variation | DF | F | P |
| --- | --- | --- | --- |
| **(a) Coral Cover** |  |  |  |
| **Forereef** |  |  |  |
| Year (fixed) | 25 | 32.93 | < 0.0001 |
| 2005 a |  |  |  |
| 2006 a |  |  |  |
| 2007 a |  |  |  |
| 2008 b |  |  |  |
| 2009 c |  |  |  |
| 2010 c |  |  |  |
| Site (random) | 25 | 3.70 | 0.0121 |
| **Backreef** |  |  |  |
| Year (fixed) | 25 | 0.49 | 0.7839 |
| Site (random) | 25 | 7.00 | 0.0003 |
| **Fringing reef** |  |  |  |
| Year (fixed) | 25 | 0.85 | 0.5278 |
| Site (random) | 25 | 44.47 | < 0.0001 |
| **(b) Macroalgae Cover** |  |  |  |
| **Forereef** |  |  |  |
| Year (fixed) | 25 | 13.45 | < 0.0001 |
| 2005 b |  |  |  |
| 2006 b |  |  |  |
| 2007 b |  |  |  |
| 2008 a |  |  |  |
| 2009 a |  |  |  |
| 2010 b |  |  |  |
| Site (random) | 25 | 4.77 | 0.0034 |
| **Backreef** |  |  |  |
| Year (fixed) | 25 | 3.17 | 0.0236 |
| 2005 a |  |  |  |
| 2006 a |  |  |  |
| 2007 a |  |  |  |
| 2008 a |  |  |  |
| 2009 a |  |  |  |
| 2010 a |  |  |  |
| Site (random) | 25 | 10.23 | < 0.0001 |
| **Fringing reef** |  |  |  |
| Year (fixed) | 25 | 2.19 | 0.0869 |
| Site (random) | 25 | 49.07 | < 0.0001 |
| **(c) Bare/CCA/Turf Cover** |  |  |  |
| **Forereef** |  |  |  |
| Year (fixed) | 25 | 20.25 | < 0.0001 |
| 2005 c |  |  |  |
| 2006 c |  |  |  |
| 2007 c |  |  |  |
| 2008 bc |  |  |  |
| 2009 b |  |  |  |
| 2010 a |  |  |  |
| Site (random) | 25 | 3.29 | 0.0202 |
| **Backreef** |  |  |  |
| Year (fixed) | 25 | 0.90 | 0.4953 |
| Site (random) | 25 | 6.09 | 0.0008 |
| **Fringing reef** |  |  |  |
| Year (fixed) | 25 | 5.51 | 0.0015 |
| 2005 a |  |  |  |
| 2006 a |  |  |  |
| 2007 a |  |  |  |
| 2008 b |  |  |  |
| 2009 ab |  |  |  |
| 2010 ab |  |  |  |
| Site (random) | 25 | 17.81 | < 0.0001 |
