# Supplementary material for: Herbivory, Connectivity, and Ecosystem Resilience: Response of a Coral Reef to a Large-Scale Perturbation
Source: PLoS One. 2011 Aug 25;6(8):e23717. doi: 10.1371/journal.pone.0023717 (PMC3162008; doi:10.1371/journal.pone.0023717)
Supplement: Table S2 — Results of mixed-effects ANOVA on the (a) density and (b) biomass of herbivorous fish in each of the three habitat types. Results of post hoc Tukey tests for the fixed effect of year are indicated; years not sharing the same letter are significantly different at P<0.05. (DOC) [file pone.0023717.s008.doc]

Table S2. Results of mixed-effects ANOVA on the (a) density and (b) biomass of herbivorous fish in each of the three habitat types. Results of post hoc Tukey tests for the fixed effect of year are indicated; years not sharing the same letter are significantly different at P < 0.05.

| Source of variation | DF | F | P |
| --- | --- | --- | --- |
| **(a) Density Roving Herbivorous Fishes** |  |  |  |
| **Forereef** |  |  |  |
| Year (fixed) | 68 | 13.74 | < 0.0001 |
| 2006 b |  |  |  |
| 2007 b |  |  |  |
| 2008 b |  |  |  |
| 2009 a |  |  |  |
| 2010 a |  |  |  |
| Transect (random) | 68 | 4.75 | < 0.0001 |
| Site (random) | 12 | 2.11 | 0.1338 |
| **Backreef** |  |  |  |
| Year (fixed) | 68 | 1.59 | 0.1879 |
| Transect (random) | 68 | 3.37 | 0.0007 |
| Site (random) | 12 | 4.66 | 0.0135 |
| **Fringing reef** |  |  |  |
| Year (fixed) | 68 | 4.04 | 0.0053 |
| 2006 ab |  |  |  |
| 2007 b |  |  |  |
| 2008 a |  |  |  |
| 2009 ab |  |  |  |
| 2010 a |  |  |  |
| Transect (random) | 68 | 4.85 | < 0.0001 |
| Site (random) | 12 | 6.91 | 0.0030 |
| **(b) Biomass Roving Herbivorous Fishes** |  |  |  |
| **Forereef** |  |  |  |
| Year (fixed) | 20 | 22.75 | < 0.0001 |
| 2006 b |  |  |  |
| 2007 b |  |  |  |
| 2008 b |  |  |  |
| 2009 a |  |  |  |
| 2010 a |  |  |  |
| Site (random) | 20 | 9.1 | 0.0001 |
| **Backreef** |  |  |  |
| Year (fixed) | 20 | 0.99 | 0.4368 |
| Site (random) | 20 | 3.32 | 0.0241 |
| **Fringing reef** |  |  |  |
| Year (fixed) | 20 | 2.61 | 0.0667 |
| Site (random) | 20 | 8.87 | 0.0001 |
