# Supplementary material for: Herbivory, Connectivity, and Ecosystem Resilience: Response of a Coral Reef to a Large-Scale Perturbation
Source: PLoS One. 2011 Aug 25;6(8):e23717. doi: 10.1371/journal.pone.0023717 (PMC3162008; doi:10.1371/journal.pone.0023717)
Supplement: Table S4 — Results of mixed-effects ANOVA on the density of parrotfishes in each of the three habitat types. Results of post hoc Tukey tests for the fixed effect of year are shown; years not sharing the same letter are significantly different at P<0.05. (DOC) [file pone.0023717.s010.doc]

Table S4. Results of mixed-effects ANOVA on the density of parrotfishes in each of the three habitat types. Results of post hoc Tukey tests for the fixed effect of year are shown; years not sharing the same letter are significantly different at P < 0.05.

| Source of variation | DF | F | P |
| --- | --- | --- | --- |
| **Density of parrotfishes** |  |  |  |
| **Forereef** |  |  |  |
| Year (fixed) | 20 | 16 | < 0.0001 |
| 2006 b |  |  |  |
| 2007 b |  |  |  |
| 2008 b |  |  |  |
| 2009 a |  |  |  |
| 2010 a |  |  |  |
| Site (random) | 20 | 7.72 | 0.0003 |
| **Backreef** |  |  |  |
| Year (fixed) | 20 | 1.63 | 0.2062 |
| Site (random) | 20 | 24.14 | < 0.0001 |
| **Fringing reef** |  |  |  |
| Year (fixed) | 20 | 2.09 | 0.1205 |
| Site (random) | 20 | 5.07 | 0.0037 |
